# Relationship between Occlusal Force Distribution and the Activity of Masseter and Anterior Temporalis Muscles in Asymptomatic Young Adults

**DOI:** 10.1155/2013/354017

**Published:** 2012-12-05

**Authors:** Aneta Wieczorek, Jolanta Loster, Bartlomiej W. Loster

**Affiliations:** ^1^Department of Dental Prosthetics, Institute of Dentistry, Jagiellonian University, 31-155 Kraków, Poland; ^2^Department of Orthodontics, Institute of Dentistry, Jagiellonian University, 31-155 Kraków, Poland

## Abstract

Healthy subjects have a prevalent side on which they display higher-muscle activity during clenching. The relationship between symmetry of masseter muscle (MM) and anterior temporalis (TA) muscle activities and occlusion has been evaluated on the basis of physiological parameters. The aim of the present study was to investigate whether the symmetry of surface EMG (sEMG) activity in asymptomatic young adults is related to symmetry of occlusal contacts. *Material*. The study population consisted of seventy-two 18-year-old subjects with no temporomandibular disorder (TMD) symptoms. *Method*. All the participants underwent an sEMG recording with an 8-channel electromyograph (BioEMG III). A T-Scan III evolution 7.01 device was used to analyze the occlusal contact points. *Results*. The correlation between the activity of right (R) and left (L) TA and the percentage of occlusal contacts was assessed, but no significant differences were found between the RMM and LMM muscles. The differences in the medium values of sEMG between males and females were not statistically significant. Equilibrated muscular activity between RTA and LTA occurred when occlusal contacts reached the percentage of 65% on the left side. *Conclusion*. The symmetry of sEMG activity in asymptomatic young adults is not related to symmetry of occlusal contacts.

## 1. Introduction

The stomatognathic system (SS) is responsible for such functions as speech, chewing, and swallowing, which remain in equilibrium in healthy individuals. Dental treatment should be planned on the basis of history taking followed by a standardized physical examination. If any abnormalities are detected within the SS, it is important to perform an in-depth analysis for additional information [1]. This process may be significantly enhanced through the use of techniques that objectively measure SS function and thereby reduce reliance on the subjective assessment of clinical observations. Rational treatment not only requires a good understanding of the pathogenesis of the diseases concerned but should also be based on an accurate diagnosis. It has been repeatedly shown that clinical examination alone can lead to gross errors in diagnosis. Temporomandibular disorders (TMDs) most often manifest with a muscular abnormality which can be analyzed by surface electromyography (sEMG) [2], but Suvinen and Kemppainen concluded that until electromyographic measures are correlated with other multidimensional, especially subjective and pain-related methods, the clinical use of this method for the diagnosis of TMD is not at present recommended [3]. Also Klasser and Okeson in their comprehensive review produced results which show that the clinical use of sEMG in the diagnosis and treatment of TMD is of limited value [4]. On the other hand, the data from other researchers lacked comparative control data, had small sample sizes, and differences in selection criteria and methodology [5–7]. According to the critical review of Suvinen and Kemppainen, a well-controlled clinical EMG study, homogeneous in subject age, is required [3].

The aim of the present study was to carry out such a study by investigating whether symmetry of sEMG activity in asymptomatic young adults is related to symmetry of occlusal contacts.

The null hypothesis assumes that there is a symmetry of sEMG activity of the masseter and anterior temporalis muscles, which is strictly dependent on symmetry of occlusal contacts as measured by the distribution of forces in relation to the maximum force exerted.

## 2. Material

The study population consisted of 18-year-old subjects (44 females and 28 males, total 72) who were invited to participate in MNiSW project no. N N403 589139 aimed at evaluating the status of the SS in healthy young individuals. All the subjects were examined clinically by the same trained dentist and answered the Polish version of the RDC/TMD questionnaire for TMD [8]. The subjects were selected from two high schools in Krakow and were qualified for the study only if they had no past contact with either of the researchers involved in the investigation or the instruments under investigation. These inclusion criteria were set to avoid any potential bias resulting from preconceived ideas [1]. The study was initiated after the subjects had signed informed consent forms, and the research program had been approved by the Ethical Committee of Jagiellonian University KBET//89B/2009. It was conducted in accordance with the *Declaration of Helsinki*.

Exclusion criteria were as follows: periodontal pathology, pain, bleeding and/or 3 mm probing depth, caries or damaged dental tissues, fixed restorations, past or ongoing orthodontic therapy, bruxism diagnosed on the basis of parafunctional facets and/or anamnesis of parafunctional tooth clenching and/or grinding, neuropathic conditions evaluated, systemic and/or localized maxillofacial disease, botox therapy, psychological disorders, and pregnancy [9].

The inclusion criteria were as follows: a full dental arch, no symptoms of TMD based on an RDC/TMD examination, and compatible occlusal and skeletal classes.

## 3. Method

All the study participants underwent an sEMG recording with a commercially available device—an 8-channel electromyograph (BioEMG III)—BioPAK Measurement System (BioResearch, Inc., Milwaukee, WI, USA). Surface EMG signals were obtained from four of the 8 channels. A T-Scan III evolution 7.01 device (Tekscan Inc., South Boston, Ma, USA) was used to analyze occlusal contact points. The instrument was directly interfaced with a computer which presented the data on a screen during the examination and recorded them for further analysis. Additionally, T-Scan III/BioEMG Integration Software (Tekscan Inc/BioResearch Associates technology partnership) was applied. This integrates the clinical data from the T-Scan III with the electromyographic data of the BioResearch's EMG unit with which it is compatible. This combination of systems makes it possible to record simultaneously the force, timing, and balance of both the craniofacial muscles and the occlusion.

All examinations were performed between 8 am and 10 am, just before school. Prior to the examination, the volunteers were seated for 5 minutes in a quiet place listening to relaxing music. The recordings were made in a quiet environment, and external noises were controlled to avoid artifacts caused by smiling or other facial expressions. The subjects were informed about the aim of the test so that they could offer the maximum cooperation. During all examinations, the patient was instructed to sit upright on a chair with the head unsupported, with the trunk perpendicular to the floor, both feet on the floor, hands resting on the lap, and looking forward. After the skin was cleaned with 95% alcohol and rubbed with abrasive paper, to reduce electrode-skin impedance, the recording was performed by the use of bipolar surface electrodes (BioFLEX: BioResearch Associates Inc., Brown Deer, WI, USA), following the manufacturer's protocol. The electrodes were placed bilaterally on the subject's skin overlying the anterior temporalis, vertically along the anterior muscular margin, approximatively over the coronal suture. For the masseter, the electrodes were placed parallel to muscular fibers, with the upper pole of the electrode at the intersection between the tragus-labial commissure and the exocanthion-gonion lines, perpendicular to the skin surface, according to the technique described by Ferrario and Sforza [10]. A plate ground electrode was secured to the forehead [11]. After the set of sEMG electrodes was positioned, the subject was invited to clench the teeth as hard as possible, three times for 3 s, with 3 s relaxation between each clench [12]. All the registrations were repeated three times. The sEMG examination involved recording the occlusion with the use of T-Scan III device. All sEMG and T-Scan measurements were made by one investigator with expertise in the use of such devices and under continued in-house training organized by the manufacturer [1]. 

The T-Scan III software automatically chose the still image with the maximal occlusal contact from the first clench of selected second registration. The value of the maximal voltage obtained in the registration of maximal clenching was analyzed. The T-scan III software automatically calculated the distribution of occlusal forces, taking 100% of the value at the maximum point of distribution of force in occlusal contacts of an individual during the test. The software used allows the maximum occlusal contact and its distribution to the right and left sides in relation to the midline to be displayed. The study protocol assumed homogeneous analysis of the left side, where the values of <50% mean occlusion are on the right side and >50% on the left side. The T-Scan III/BioEMG Integration Software showed maximum voltage of muscles for this moment, bringing data up from the still of T-Scan registration. 

After the sEMG and T-scan examinations, alginate impressions (Kromopan-Zhermapol, Poland) were taken, and cephalometric radiographs were taken using the ProMax radiographic unit (Planmeca, Finland, 2005).

The analysis of occlusal and skeletal classes was made by means of plaster study models and cephalometric examinations. The cephalometric findings were analyzed using the Kracovia Composite System (based on Bjork cephalometry) [13]. For assessing the sagittal jaw relationship, we used the ANB angle, which was verified by Wits measurements, and jaw length (maxilla and mandible to porion). Jaw length was recorded as a percentage ratio, and this value was used in some doubtful (borderline) cases. 

Measurement variability was assessed by repeated sEMG analyses of two subjects chosen at random. The operator performed three independent sessions on the following days at this same hour each day and using the same protocol, namely, that described by Ferrario et al. [14]. A recording lasting 2000 ms was made in each test from the first to the third second. Accuracy and precision were calculated by means of the protocol used by Ferrario et al., namely, intraclass correlation coefficient (ICC) analysis [7].

Data analysis involved the following steps.

1. Measurement variability.
2. Occlusal and skeletal classes of subjects.
3. The sEMG values in relation to the gender.
4. SEMG activity.
5. Occlusal contacts distribution.
6. Relationship between occlusal force distribution and sEMG activity.
7. Division into two groups: more than 50% and less than 50% in relation to the left side.
8. Division into three groups: distribution of force in occlusal contacts almost equal to 50% (50 ± 0.5%), higher than 50.5%, and lower than 49.5% in relation to the left side.
9. Division into three groups: first with distribution of force in occlusal contacts of  50 ± 5%, the second higher than 55%, and the third lower than 45% in relation to the left side.

## 4. Statistical Analysis

All the data were analyzed using the SPSS (Statistical Package for the Social Sciences) Statistics 17.0 (2008) for Windows. Data normality was tested using the Kolmogorov-Smirnov (with Lilliefors correction) and Shapiro-Wilk tests. For nonnormal data, the nonparametric Mann-Whitney and Kruskal-Wallis tests were used. For normal distribution, the Student's *t*-test was used. Intraclass correlation coefficient (ICC) was tested using *F*-test. Statistical significance was set at 5% (*P* < 0.05).

## 5. Results

1. Measurement variability was assessed by repeated sEMG analyses of three subjects and tested by computing the ICC. For all sEMG variability, the ICC was 0.765, showing a good accuracy of the measurements (as shown in Table 1).
2. Occlusal and skeletal classes of subjects. Using the methodology described above, classes were as follows: 46 subjects were of class I, 19 of class II, and 7 of class III.
3. The sEMG values in relation to the gender. As presented in Table 2, the differences between males and females in medium values of sEMG for the right temporalis anterior (RTA), left temporalis anterior (LTA), right masseter (RMM), and left masseter (LMM) muscles were not statistically significant. Therefore, all subsequent calculations were performed regardless of gender.
4. sEMG activity. Surface EMG recordings from the left and right masseter and anterior temporalis muscles demonstrate statistically significant differences in voltage of the muscles (*P* = 0.03) (Table 3), but no significant differences between LMM and RMM muscles (*P* = 1.00).
5. Occlusal contacts distribution. The normality of data was analyzed using the Kolmogorov-Smirnov (with the Lilliefors correction, *P* = 0.2) and the Shapiro-Wilk (*P* = 0.236). Due to insignificances, the normal distribution of results was analyzed by the use of the Student's *t*-test (*P* < 0.05).

In the analysis of the percentage of occlusal force, there were no significant differences observed between females and males (Table 4). However, the results showed that in female population, the percentage of occlusal force distribution on the left side was approximately 52% in comparison to 48% in male population. This difference is not statistically significant.

**Figure 1:**
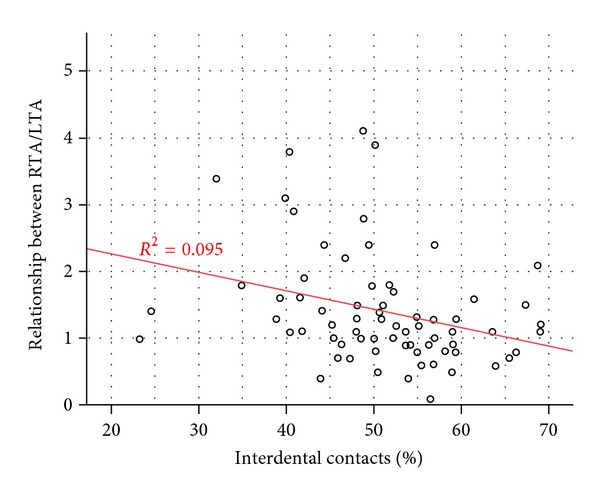
Relationship between RTA and LTA and distribution of force in occlusal contacts presented as a percentage in relation to the left side. This line is the regression equation; the ratio of RTA/LTA = 2.81 (constant beta) − 0.027 (beta for interproximal contacts) ∗ interproximal contacts. This line shows the direction of the relationship, with an increase in contacts between the teeth, reducing RTA to LTA ratio. The *R* squared = 0.95 means that the change in the value of “interproximal contacts (left%)” at 9.5% is based on changes in the value of “the ratio of RTA/LTA.”

(6) Relationship between occlusal force distribution and sEMG activity. The correlation between the activity of the right and left anterior temporalis muscles (*y*) and the percentage of occlusal contacts (*x*) was assessed. As presented in Figure 1, when occlusal contacts reached the percentage of 65% on the left side, equilibrated muscular activity between RTA and LTA occurred. A value above 65% indicated increased activity of the left temporalis anterior muscle.

The normality of data was tested using the Kolmogorov-Smirnov (with Lilliefors correction) and Shapiro-Wilk tests. Any nonnormal distribution of results was subjected to further analysis using parametric Mann-Whitney and Kruskal-Wallis tests. Statistical significance was set at 5% (*P* < 0.05). In the next step, any correlation between the activity of right and left temporalis anterior muscles and the percentage of occlusal contacts was assessed. For this purpose, the results were divided into two groups; those with more than 50% contacts (*n* = 39) and those with less than 50% (*n* = 33).

(7) Division into two groups: more than 50% and less than 50% in relation to the left side. As shown in Table 5, the percentage of occlusal contacts was higher on the right side in the group with interdental contact values <50%. The mean value of differences between RTA and LTA in the group with a distribution of forces in occlusal contacts <50% was significantly higher (*P* = 0.005). This is also clearly shown in the data presenting the relationship between RTA and LTA. On the other hand, when the dominance of occlusal contacts was on the left side, the right-sided dominance of the muscle activity was lower, but still significant (*P* = 0.002). This relation was not significant between left and right masseters.

Table 6 shows that the percentage of occlusal contacts was higher on the right side in the group with interdental contact values <50%. The mean value in differences between RMM and LMM showed a preponderance of right over left masseter muscles and, in the group with a distribution of force in occlusal contacts >50%, a preponderance of left over right masseter muscles. These values were not statistically significant with the relation between RMM/LMM (*P* = 0.82) and the differences between RMM and LMM (*P* = 0.84), respectively.

(8) Division into three groups: distribution of force in occlusal contacts almost equal to 50% (50 ± 0.5%), higher than 50.5%, and lower than 49.5% in relation to the left side.

For further analysis of the correlation between occlusal contacts and symmetry of activity of the temporalis anterior, a further division of data was made between occlusal contacts of 50 ± 0.5% (6 subjects), contacts >50.5% (36 subjects), and those with <49.5% contacts (30 subjects). Here, the statistical analysis involved the Kruskal-Wallis test.

The analysis concerning the percentage of occlusal contacts revealed that, in the group with force distributions equaling 50 ± 0.5%, right temporalis anterior muscle activity was predominant, (Table 7) and this domination was still observed when occlusal contacts were more visible on the right side and even slightly persisted when contacts were mainly present on the left side (*P* = 0.04). The same tendency was displayed in the analysis of the relationship between RTA and LTA (*P* = 0.016).

The analysis concerning left and right masseters is shown in Table 8 in relationship to the group with force distributions >50.5% on the left side, and dominance of left masseter muscles was shown. This same tendency was seen in the group with force distribution <49.5% on the left side with slight dominance of left masseter muscles. In the group with force distributions equaling 50 ± 0.5%, dominance of the right masseter muscle was shown. All these results were statistically significant (*P* = 0.003).

(9) Division into three groups: the first with a distribution of force in occlusal contacts of  50 ± 5%, the second higher than 55%, and the third lower than 45% in relation to the left side. Finally, the data were divided as follows. The first part of the 50 ± 5% group included the examination results of 31 subjects, the second >55% part of 23 subjects, and the third <45% part of 18, respectively (Tables 9 and 10). In the first group, right temporalis anterior muscle activity prevalence was demonstrated in the relation of RTA/LTA (*P* = 0.04) and the difference between RTA and LTA (*P* = 0.012) (Table 9). Only in the group with over 55% occlusal contacts on the left side was a significant dominance of left temporalis anterior muscles activity visible. However, the mean value of the difference between RTA and LTA was lower (−4.04 ± 54.444) in comparison with the dominance of occlusal contacts on the right side (46.94 ± 52.571).

The analysis concerning left and right masseters is shown in Table 10 in relationship to the group with force distributions >55% according to the left side with dominance of left masseter muscles. In the group with force distribution <45% on the left, dominance of left masseter muscles was shown. In the group with force distributions equaling 50 ± 5%, dominance of the right masseter was shown. All these results were not statistically significant (*P* = 0.152).

## 6. Discussion

The aim of the study was to investigate whether there was any correlation between symmetry of masseter and anterior temporal muscles activities and occlusal force distribution in asymptomatic young adults. A search of the literature related to the relationship between the distribution of force in occlusal contacts and sEMG of masseter and anterior temporalis muscles did not reveal many studies involving a comparative age group. At this age (18 years), all 28 teeth are already erupted, bone growth is finished, or is almost finished and the teeth have low levels of physiological abrasion. All the subjects in our study had no prior history of orthodontic treatment or any symptoms of TMD, based on an RDC/TMD examination.

It is reasonable to believe that the homogeneity of the studied sample enhanced the internal validity of the study. As reported by other researchers, we found in our study that the voltage recorded in the anterior part of the temporalis muscle is higher than in the masseter muscle in healthy subjects. Some researchers have demonstrated asymmetry in clench potentials between left and right muscles [15]. Similarly, Suvinen and Kemppainen [3] and Scopel et al. [16], in their investigations, reported that even in the mandibular postural position, asymptomatic muscles were physiologically asymmetrical, and that asymmetry and activity indices of 4% and 17%, respectively, were to be considered as normal. Moreover, this study showed that most subjects presented a right-side temporalis anterior muscle dominance.

On the basis of our study, it may be concluded that in asymptomatic patients, asymmetry tends to affect the right side of the temporalis anterior, especially when the percentage of interdental contacts is close to 50%. However, in our study, there were few subjects with a symmetric distribution of occlusal force. As mentioned previously, Ferrario et al. [5] showed that healthy individuals have a prevalent side on which they generate higher levels of muscular activity during bilateral clenching [17]. A higher level of muscular activity of RTA was demonstrated in our study, and this tendency was still visible, even when occlusal forces were more evenly distributed on the left side.

Some studies report that a T-Scan sensor significantly influences sEMG activity of the superficial masseter muscle, whereas it does not affect the anterior temporalis when compared to occlusion in the natural dentition. Some researchers suggest that T-Scan is a good tool for chair-side analysis of the occlusion [6, 18, 19]. However, the T-scan III system can be integrated with the BioEMG III system. In this way, synchronized clinical data can be recorded simultaneously. Additionally, the measurements obtained can be comparatively reviewed during playback [20]. For the time being, T-Scan III/BioEMG Integration Software is considered as the best tool in the market since it allows simultaneous recording analysis of the sEMG of selected muscles and of the distribution of occlusal force while correlating specific occlusal moments with specific electromyographic changes.

Analysis of tension of TA and MM muscles in each analyzed occlusal force distribution shows that the right-handed tendency of TA muscles can be corrected by the left-handed asymmetry of MM muscles. However, this requires further well-designed and controlled research and analysis.

## 7. Conclusion

The null hypothesis that the symmetry of electromyographic activity of masseter and anterior temporalis muscles is strictly connected with symmetry in occlusal contacts, as measured by distribution of forces in relation to the maximum force exerted, was rejected.

The symmetry of sEMG activity in asymptomatic young adults is not related to symmetry of occlusal contacts.

## Figures and Tables

**Table 1:** Intraclass correlation coefficient (ICC).

	Intraclass correlation	95% Confidence interval		*F*-Test with true value 0			
		Lower bound	Upper bound	Value	df1	df2	*P* < 0.05
Average measurements	0.765	0.540	0.890	4.259	23	48	0.000

**Table 2:** The sEMG values for right and left masseters and temporalis anteriors in male and female members of the study group. Mann-Whitney test.

	Male (*n* = 28)				Female (*n* = 44)				*P* < 0.05
	Mean *μ*V	SD	Min *μ*V	Max *μ*V	Mean *μ*V	SD	Min *μ*V	Max *μ*V	
RTA	119.93	93.471	15	397	133.14	69.833	25	428	0.06
LTA	92.25	42.655	11	197	108.73	61.428	24	314	0.44
RMM	147.89	97.632	31	355	115.05	74.431	11	347	0.27
LMM	130.46	93.779	27	492	121.45	72.733	42	335	0.69

**Table 3:** The sEMG values for right and left masseters and temporalis anteriors. Mann-Whitney test.

	Mean *μ*V	SD	Min *μ*V	Max *μ*V	*P* < 0.05
RTA	128.00	79.485	15	428	0.03
LTA	102.32	55.160	11	314	
RMM	127.82	85.089	11	355	1.00
LMM	124.96	81.042	27	492	

**Table 4:** The percentage of force of occlusal contacts in male and female members of the study group. Student's *t*-test.

	Gender	*N *	Mean %	SD	*P* < 0.05
Interdental contact (left%)	Males	28	48.2	9.4	0.074
	Females	44	52.3	9.2	

**Table 5:** Results for interdental contacts, in percentages, with the division into more than 50% and less than 50% in relation to the left side for temporalis anterior muscles. Mann-Whitney test.

Interdental contacts in %		*N *	Mean	SD	Min	Max	*P* < 0.05
Relationship between RTA and LTA	Left (>50%)	39	1.146	0.6468	0.1	3.9	0.002
	Right (<50%)	33	1.739	0.9344	0.4	4.1	

Differences between RTA and LTA	Left (>50%)	39	5.38 *μ*V	68.518	−145 *μ*V	268 *μ*V	0.005
	Right (<50%)	33	49.67 *μ*V	73.495	−79 *μ*V	299 *μ*V	

**Table 6:** Results for interdental contacts, in percentages, with the division into more than 50% and less than 50% in relation to the left side for masseter muscles. Mann-Whitney test.

Interdental contacts in %		*N *	Mean	SD	Min	Max	*P* < 0.05
Relationship between RMM/LMM	Left (>50%)	39	1.059	0.4924	0.1	2.3	0.82
	Right (<50%)	33	1.171	0.6373	0.5	3.2	

Differences between RMM and LMM	Left (>50%)	39	−3.09 *μ*V	70.700	−199 *μ*V	132 *μ*V	0.84
	Right (<50%)	33	9.92 *μ*V	69.923	−137 *μ*V	217 *μ*V	

**Table 7:** Results for interdental contacts, presented in percentages, with data divided into three groups: 50 ± 0.5%, more than 50.5%, and less than 49.5% in temporalis anterior muscles. Kruskal-Wallis test.

Interdental contacts in %		*N *	Mean	SD	Min	Max	*P* < 0.05
Relationship between RTA and LTA	Left (>50.5%)	36	1.097	0.4687	0.1	2.4	0.016
	Right (<49.5%)	30	1.740	0.9640	0.4	4.1	
	50 ± 0.5%	6	1.733	1.2707	0.5	3.9	

Differences between RTA and LTA	Left (>50.5%)	36	3.17 *μ*V	49.452	−127 *μ*V	126 *μ*V	0.04
	Right (<49.5%)	30	44.90 *μ*V	71.096	−79 *μ*V	299 *μ*V	
	50 ± 0.5%	6	64.67 *μ*V	152.101	−145 *μ*V	268 *μ*V	

**Table 8:** Results for interdental contacts, presented in percentages, with data divided into three groups: 50 ± 0.5%, more than 50.5%, and less than 49.5% in masseter muscles. Kruskal-Wallis test.

Interdental contacts in %		*N *	Mean	SD	Min	Max	*P* < 0.05
Relationship between RMM and LMM	Left (>50.5%)	36	1.020	0.4909	0.1	2.3	0.016
	Right (<49.5%)	30	1.114	0.6395	0.5	3.2	
	50 ± 0.5%	6	1.635	0.1996	1.4	1.9	

Differences between RMM and LMM	Left (>50.5%)	36	−9.29 *μ*V	69.157	−199 *μ*V	132 *μ*V	0.003
	Right (<49.5%)	30	−1.12 *μ*V	63.064	−137 *μ*V	217 *μ*V	
	50 ± 0.5%	6	95.83 *μ*V	42.537	31 *μ*V	134 *μ*V	

**Table 9:** Interdental contacts in percentages, with data division into three groups: 45%–55%, more than 55%, and less than 45%. Kruskal-Wallis test.

Interdental contacts in %		*N *	Mean	SD	Min	Max	*P* < 0.05
Relationship between RTA and LTA	Left (>55%)	23	1.052	0.5125	0.1	2.4	0.004
	Right (<45%)	18	1.872	0.9336	0.4	3.8	
	45%–55%	31	1.426	0.8706	0.4	4.1	

Difference between RTA and LTA	Left (>55%)	23	−4.04 *μ*V	54.444	−127 *μ*V	126 *μ*V	0.012
	Right (<45%)	18	46.94 *μ*V	52.571	−79 *μ*V	166 *μ*V	
	45%–55%	31	35.39 *μ*V	89.949	−145 *μ*V	299 *μ*V	

**Table 10:** Results for interdental contacts, in percentages, with data divided into three groups: 45%–55%, more than 55%, and less than 45%. Kruskal-Wallis test.

Intedental contacts in %		*N *	Mean	SD	Min	Max	*P* < 0.05
Relationship between RMM and LMM	Left (>55%)	23	1.031	0.4320	0.2	2.1	0.267
	Right (<45%)	18	1.014	0.5623	0.5	2.1	
	45%–55%	31	1.226	0.6385	0.1	3.2	

Differences between RMM and LMM	Left (>55%)	23	−3.88 *μ*V	63.938	−199 *μ*V	132 *μ*V	0.152
	Right (<45%)	18	−14.81 *μ*V	56.789	−137 *μ*V	102 *μ*V	
	45%–55%	31	18.16 *μ*V	79.737	−180 *μ*V	217 *μ*V	
